# Comparative Analyses of Medicinal Chemistry and Cheminformatics Filters with Accessible Implementation in Konstanz Information Miner (KNIME)

**DOI:** 10.3390/ijms23105727

**Published:** 2022-05-20

**Authors:** Sebastjan Kralj, Marko Jukič, Urban Bren

**Affiliations:** 1 Laboratory of Physical Chemistry and Chemical Thermodynamics, Faculty of Chemistry and Chemical Engineering, University of Maribor, Smetanova 17, 2000 Maribor, Slovenia; sebastjan.kralj1@um.si; 2 Faculty of Mathematics, Natural Sciences and Information Technologies, University of Primorska, Glagoljaška 8, 6000 Koper, Slovenia

**Keywords:** high-throughput virtual screening, virtual screening, compound libraries, library design, compound filtering

## Abstract

High-throughput virtual screening (HTVS) is, in conjunction with rapid advances in computer hardware, becoming a staple in drug design research campaigns and cheminformatics. In this context, virtual compound library design becomes crucial as it generally constitutes the first step where quality filtered databases are essential for the efficient downstream research. Therefore, multiple filters for compound library design were devised and reported in the scientific literature. We collected the most common filters in medicinal chemistry (PAINS, REOS, Aggregators, van de Waterbeemd, Oprea, Fichert, Ghose, Mozzicconacci, Muegge, Egan, Murcko, Veber, Ro3, Ro4, and Ro5) to facilitate their open access use and compared them. Then, we implemented these filters in the open platform Konstanz Information Miner (KNIME) as a freely accessible and simple workflow compatible with small or large compound databases for the benefit of the readers and for the help in the early drug design steps.

## 1. Introduction

Combinatorial chemistry (CC), novel library design methodologies, and high-throughput screening (HTS) represent the standard approaches for synthesis and evaluation (searching and selecting) of potential lead compounds in drug design efforts [1]. The combined use of chemical libraries and HTS to sift through large libraries and select desired compounds vastly increases the success rate of drug discovery programs [2,3]. Assays are now performed with libraries consisting of several million compounds: (Pfizer, 4 million [4]; Novartis 1.7 million [5]; Astra Zeneca 4 million [6]). Physical compound libraries and HTS are still regarded as the staple method for identification of leads; however, the advance of computational tools and in silico chemistry means that computer-aided methods have become indispensable in modern drug design efforts. If commercial physical compound libraries include several million molecules, the virtual compound libraries nowadays span from 10^7^ to 10^18^ molecules. Nevertheless, such expansion of chemical space is a double-edged sword, as on one hand the probability of finding potential leads when screening larger libraries is greater, but on the other hand, screening of entire libraries even with the aid of computational methods may not be economically viable or even accessible in a timely manner. With the identification of the biological target in the early steps of the drug discovery process and the definition of the binding site, the chemical space adequate for further lead design becomes specific, and such information can be used to tailor compound libraries [7]. The specific nature of molecular recognition and interaction combined with the fact that drugs must exhibit additional properties such as bioavailability and acceptable toxicity profiles severely narrows the adequate chemical space making drug design a monumental undertaking. Therefore, in a contemporary VS (virtual screening) or HTVS (high-throughput virtual screening) scenario, the database design is essential for efficient downstream calculations and in vitro testing. In order to achieve success in drug design efforts we need to adhere to certain library design guidelines. Libraries should focus the chemical space on the specific problem at hand, the compounds synthesizable and enriched with molecules that have drug-like properties [8].

The main challenge of library construction is to cover as much diversity of the chemical space as possible, while keeping the total number of compounds low to reduce time and money consumption [9,10]. Various molecular filters are often used to increase the hit rates of drug development campaigns [11]. With the hit rate of screening being on average as low as 1%, the simplest and most direct way to increase hit rate is to eliminate molecules with a low probability of becoming leads [12]. Filtering removes both unwanted chemical structures and unwanted chemical properties and is used to tailor the molecular libraries in a target focused manner [1]. The work on molecular filters was pioneered by Chris Lipinski and coworkers, who compared early HTS and combinatorial chemistry drug hits at Pfizer (up to 1994) with a subset of 2245 drugs from the World Drug Index [13]. The aim was to understand the common molecular features of orally available drugs and using an efficient version of the QSAR paradigm for structure permeability as suggested by Van de Waterbeemd et al. [14]. They came to several conclusions on the factors affecting poor absorption and permeation [15]. The main principle behind filtering of libraries is based on the term of drug-likeness. Although the term is often used in different ways by different authors, it generally refers to molecules that have properties or contain functional groups that are consistent with the majority of the known drugs [3,16,17]. The typical drug-like compounds exhibit desirable properties such as oral bioavailability, low toxicity, membrane permeability, and reasonable clearance rates [18]. Drug-like molecules therefore occupy distinct chemical space described by molecular descriptors and assigned cut-off values derived from experience. The first and to this day the most popular filters in use focused on finding effective and orally absorbable compounds [3]. The main goal of such filters was to address ADME (absorption, distribution, metabolism, and excretion) issues. The research on this topic points towards the fact that certain properties such as logP, MW (molecular weight), and number of hydrogen bonding groups correlate with oral bioavailability. This fact has been used to improve the success of finding lead-like molecules with filters that bias the chemical space of libraries, resulting in filters designed for various drug development applications [16,17]. Besides filters for drug-like properties, several filters exist that adopt the same knowledge-based approach in their design but expand beyond the scope of classic drug-like filtering. Filters such as the Ro4 (rule-of-4), designed to focus libraries on protein–protein interaction inhibitors, use descriptor cut-offs that are opposite of what is traditionally defined as drug-like and attest to the universal nature of molecular filters [19].

With preparation of molecular libraries, it is not just a question of what to filter out but when. Rules in the form of filters mean that compounds are discriminated on a pass or fail basis—compounds that pass the rules are considered equal, as are all that breach the rules [20]. Typically, filters are employed in the starting steps of a drug discovery campaign. Applying such filters upfront reduces the number of compounds analyzed in successive steps, speeding up the drug development process. However, this comes at the price of eliminating compounds that could show desirable properties in later phases. This is especially true for stringent filters [21] and for the use of compounds that have conformational flexibility [22]. The application of filters in the later stages avoids the problem of eliminating potential leads, but also causes the computationally intensive tasks to be performed on larger libraries, increasing both the financial and time costs. Moreover, we would like to point out that some authors argue against screening out promiscuous compounds in the early drug discovery [23]. Opponents of filtering point out that any rule-based system of filtering ignores the fact that exceptions exist, and that blind use of such restrictive filters would eliminate potential drugs such as cyclosporine and erythromycin, where the majority of the drug-like rules break down [3]. Exceptions such as the aforementioned drugs bring up an important topic of distinction between properties of useful lead-like molecules and drugs. Regardless of whether the screening is done upfront of filtering on more diverse libraries or after filtering on more focused libraries, structural changes for lead optimization will usually be necessary [3]. In general structures, lead compounds exhibit less molecular complexity (less MW, fewer number of rings and rotatable bonds) and are less hydrophobic (lower clogP and logD). This indicates that the process of optimizing simple leads into drugs is favorable, supporting the idea of filtering libraries before screening and optimizing them into drugs later [24]. Filtering out “undesirable” molecular species using computational filters thus forms a key element in library preparation and carries an informed decision in defining “favorable” or “undesirable” properties [25]. Thresholds for such properties are often derived from the experience of the pharmaceutical industry [21]. The criteria of “undesirable” structures should always be considered in their suitable scientific context, e.g., the loss of peptidomimetic molecules employing typical rule-based filters such as the Ro5 (Lipinski’s rule of five) in the development of a protease inhibitor library would result in a poor hit rate [13,26]. Therefore, we encourage the reader to consider the biological context of the target, the drug discovery campaign, and to employ a plethora of filters to flag compounds for consideration and design in the subsequent drug discovery campaign steps. When using multiple filters in a sequential manner it is generally best to employ the filter that removes the most compounds first to reduce time consumption in later steps. One should also consider which filters will be applied without exceptions and which ones will merely flag the compounds for later assessment. Those that will filter without exceptions should be applied beforehand. A good example of a consecutive filtering protocol is described in the work of Jukič et al., where the library was first filtered for large and small compounds followed by filtering for aggregators, PAINS and REOS [27].

To successfully apply filters in HTVS, the selected compound library must use supported data formats, for example, the string representation SMILES (simplified molecular input line entry specification format) or 3D representations such as SDF (structure-data file format) or MOL (MDL Molfile) [28]. In most cases, 3D conformational data are not required for the use of filters, as these filters are usually referred to as “2D filters”. Despite the widespread adoption of SMILES for storage and interchange of chemical structures no standard for generating SMILES strings exist. The application of canonical SMILES, which use only a single string per molecule, is recommended to avoid duplication and problems in future filtering. To address issues of specifying isotopism and stereochemistry of a molecule the isomeric SMILES was developed and is useful for scoping the library for stereoisomerism duplicates or to generate stereoisomers and expand the chemical space. A SMILES string can be canonical and isomeric at the same time [29]. The SMILES expansion SMARTS (SMILES arbitrary target specification) allows specification of sub-structural patterns and is used for specification of protonation state, hydrogen count, and ionization states. As both the SMILES and SMARTS format are not an open project and are proprietary, this has resulted in the use of different generation algorithms by software developers, resulting in different SMILES versions for the same compounds. Moves towards the open-source string representations of compounds and standardization have been made with OpenSmiles and InChI [30]. However, with the current state of compound libraries the use of standardized chemical forms is not the norm, and care should be taken when combining such libraries for virtual screening [10]. We recommend the use of Konstanz Information Miner (KNIME) software for standardizing the input format before filtering either from the 3D SDF or the string SMILES representation, in an analogous way performed in the filters provided by this article.

Many filters for compound library design are present in primary scientific literature with some such as Lipinski’s rule-of-5 enjoying widespread recognition in the scientific community; however, many filters for drug design do not enjoy the same recognition. To bridge the gap between molecular filters and their accessibility to the public, we sought out to implement them in an open-access program that allows visual and dataflow programming through a graphical user interface. We therefore collected data on molecular filters, implemented them into existing open-access software, and compared them side by side to benefit the reader in his/her early drug design steps [31].

## 2. Results

**Figure 1:**
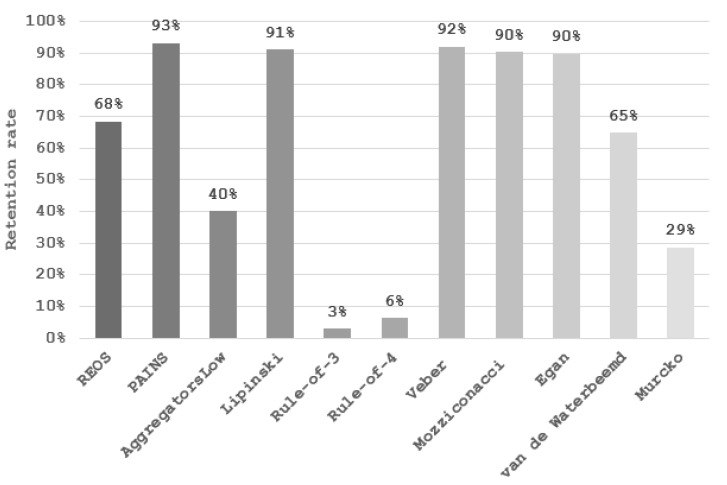
Percentage of compounds passing the described filters. The total number of sampled compounds of the unfiltered library is used as the denominator.

**Figure 2:**
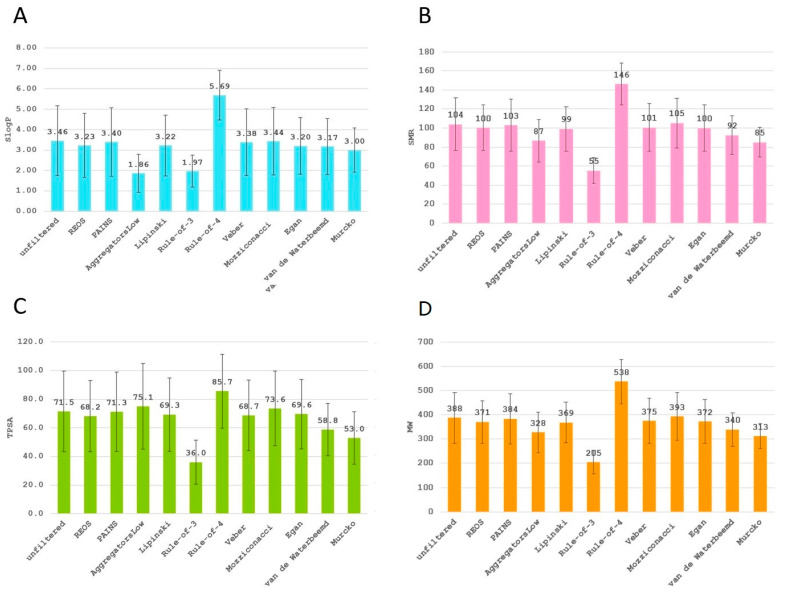
(**A**) The average descriptor value with SD of SlogP for the unfiltered and post filtering libraries. The majority of the filters have values close to the average, with AggregatorsLow and Rule-of-3 scoring lower, since they have strict cut-off values for SlogP. The partition coefficient is used to assess the lipophilicity of a drug and its ability to cross cell membranes. (**B**) The average descriptor value with SD of SMR for the unfiltered and post filtering libraries. The clear outliers are the Ro3 and Ro4, which strictly define the chemical space. (**C**) The average descriptor value with SD of TPSA for the unfiltered and post filtering libraries. The Ro3 with molecules small in size scores lower than the average, with the Ro4 being slightly above the average, but not as significantly as in the previous graphs. (**D**) The average descriptor value with SD of MW for the unfiltered and filtered libraries. The molecular weight descriptor is a very common descriptor used for cut-off values. As most of the filters aim at drug-like molecules except for the Ro4 and Ro3, the average weights are very similar.

**Figure 3:**
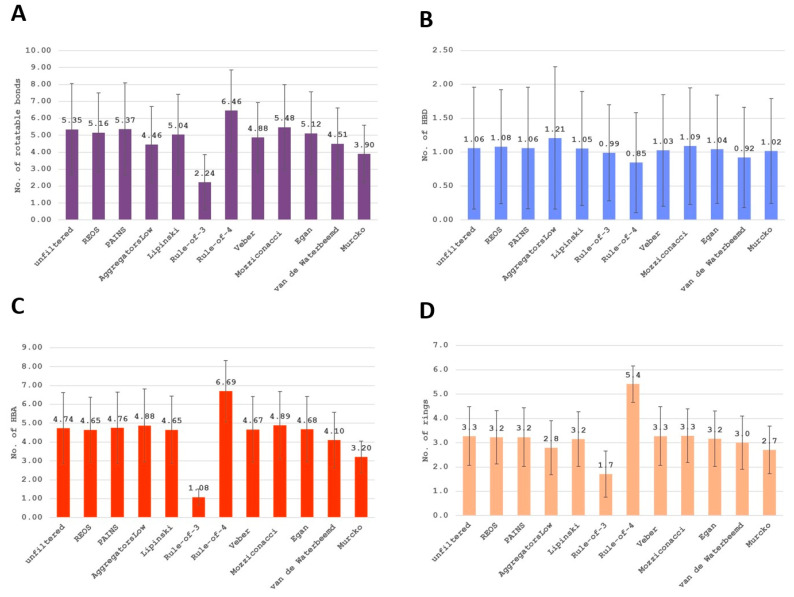
(**A**) The average descriptor value with SD of the No. of rotatable bonds for the unfiltered and filtered libraries. The graph bears resemblance to the other graphs where Ro3 and Ro4 stand out, with the other filters having average values close to the unfiltered library. (**B**) The average descriptor value with SD of the No. of HBD. We see that molecules that pass the aggregators filter have a slightly higher value of hydrogen bond donors. What is interesting is also the fact that the Ro4 scores lower than the average, despite having molecules that are larger and contain more N and O atoms which are usually involved in hydrogen bonding (Figure 4). (**C**) The average descriptor value with SD of the No. of HBA before and after filtering of the library. The Ro3 filter has a significantly lower value as its aim is to find the starting fragments from which the molecule is built. This usually leaves space for the attachment of desired functional groups to the fragment, but as a result the number of HBA is lower. (**D**) The average descriptor value with SD of the No. of rings present before and after filtering of the library. As Ro4 shifts the chemical space towards larger molecules the number of rings increases as well. The opposite happens with Ro3 where the small molecular weight does not allow for a large number of rings to be present.

**Figure 4:**
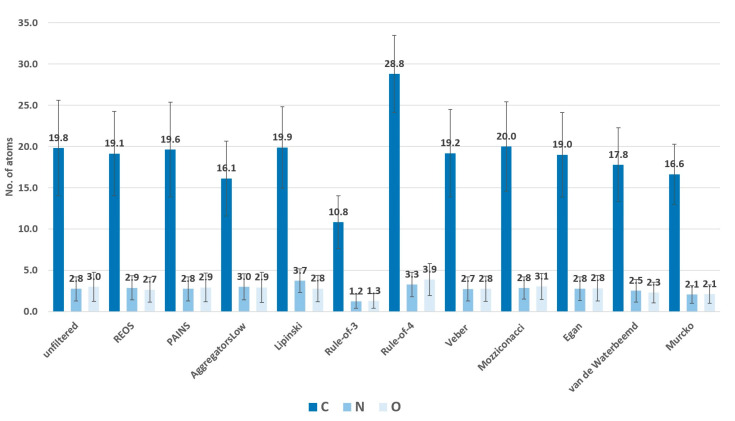
The average number of C, N, and O atoms present in the compounds. The majority of the libraries are within the average values of the unfiltered, with Rule-of-4 having higher values since the filter retains large molecules that are better suited for inhibiting protein–protein interactions. Rule-of-3 scores lower as it retains smaller compounds suitable for fragment-based drug design.

**Figure 5:**
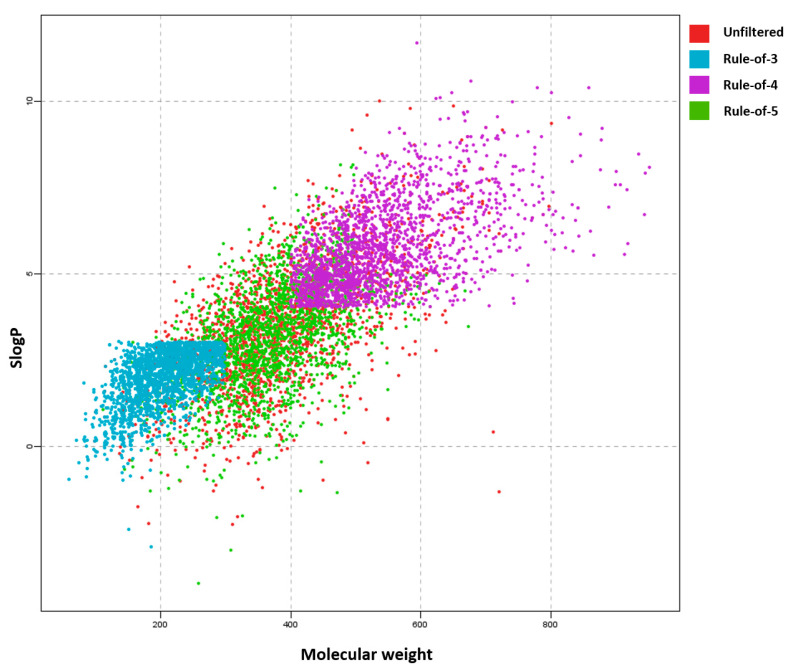
A 2D scatter plot of SlogP and molecular weight for compounds that passed individual filters. We can see the impact of using filters on the chemical space as the Rule-of-3 (blue) is totally separated from the Rule-of-4 (purple) group. This is due to the strict molecular weight cut-off and the SlogP cut-off as we see horizontal and vertical lines indicating where the cut-offs are. Since Lipinski’s Rule-of-5 (green) allows one rule break per compound, we do not observe such strict horizontal lines and the chemical space after filtering is still very similar to the unfiltered (red) library.

**Figure 6:**
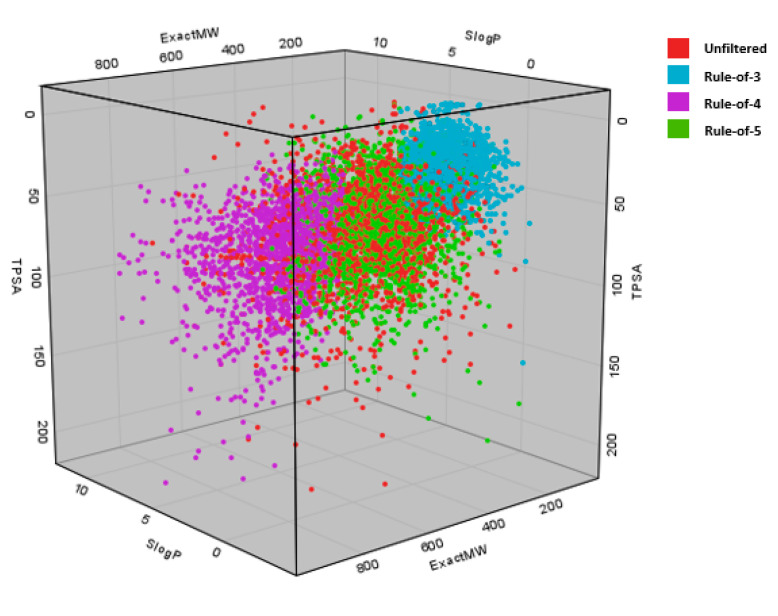
A 3D scatterplot of exact molecular weight, SlogP, and total polar surface area (TPSA) for the unfiltered (red) and filtered Rule-of-3 (Blue), Rule-of-4 (purple), and Rule-of-5 (green). We can see how the space occupied by compounds changes drastically; similar observations can be made as in the case of the 2D plot in Figure 5.

To test and demonstrate the functionality of the filters implemented and their effects on the chemical space, we applied the filtering workflow on a general ZINC database [32]. The database was obtained by accessing the ZINC website (https://zinc.docking.org/tranches/home/ accessed on 21 June 2021) selecting the following parameters (representation “2D”, reactivity “standard”, purchasability “in-stock”) and downloading the SMILES wget command file. The final downloaded library consisted of 9,216,175 compounds (a large non-specific chemical library). Using the KNIME row sampling node, 1% of the total database was sampled and ran through all the filters implemented in KNIME. We then calculated the average values and standard deviations (SD) of several key molecular descriptors using the statistics KNIME node to assess the change in chemical space after filtering (Figure 1, Figure 2, Figure 3, Figure 4, Figure 5 and Figure 6). The descriptors chosen were a standard basic set most descriptive for initial chemical space assessment; the partition coefficient as SlogP, molecular refractivity (SMR), total polar surface area (TPSA), molecular weight (MW), No. of rotatable bonds, No. of hydrogen bond acceptors (HBA), No. of hydrogen bond donors (HBD), No. of heavy atoms, No. of rings, and the number of atoms C, N, O present in the compounds. We see that filters impact the chemical space of libraries to various degrees. The more specific the filter, the larger the portion removed, since the chemical space on which they are based is far more defined than with general drug-like filters.

## 3. Discussion

The REOS (rapid elimination of swill) filter removed 32% of the compounds from the tested ZINC database subset but left the chemical space unaffected when compared to the original dataset, as it has no cutoffs on the investigated descriptors. This holds true for the functional group filter for PAINS (pan-assay interference compounds) as well; however, only 7% of compounds were removed. This is due to the fact that the filter is not as broad and tries to remove certain problematic moieties that were not captured in previously developed functional group filters (e.g., REOS). The aggregator filter, designed by Irwin et al. compares similarity of a library to 12.600 known aggregators (http://advisor.bkslab.org/rawdata/ accessed on 10 February 2022), (set to the most stringent cutoff of “low” similarity to known aggregators) removes ~60% of the database and significantly lowers the SlogP value, as it uses this descriptor as a cutoff to determine the aggregation propensity. The number of rings is also lower after filtering for aggregators implicating that the presence of rings might be involved in aggregation. However, this descriptor is not used as a cutoff, but is indirectly correlated with the properties of aggregators. The average value of rings for the dataset of known aggregators is 3.6 ± 1.03, which is slightly above the average of 3.3 ± 1.2 for the general database, meaning that compounds with rings would likely score higher in the Tanimoto coefficient comparison and get filtered out. The Ro3 (rule of three) and Ro4 (rule of four) filters are the most stringent filters as they define the most specific chemical space, filtering out 97% and 94% of the database, respectively. Despite their similarity in the filtered-out percentage, they operate in opposite ways. The Ro3 represents a strict filter designed to support “hit identification” and “fragment-based” drug research and only accepts molecules with a molecular weight of less than 300. It supports the paradigm that small compounds still capture the desired chemical space yet leave a lot of space for future compound optimization towards leads. The Ro4 attempts to capture the protein–protein interaction inhibitor chemical space and retains molecules with molecular weight above 400, as such larger molecules are able to form multiple interactions. Morelli et al. designed the filter with the aim of establishing guidelines for druggable protein–protein inhibitors, since these most often break traditional property filter rules. Beside the high MW cutoff, Ro4 retains only compounds containing multiple rings and is often above average in the descriptor value graphs (Figure 2, Figure 3 and Figure 4). The Veber and Egan filters remove a small fraction of molecules with 7.9% and 10.3%, respectively, as they both apply only two filtering rules with a mild cut-off value. The Veber filter tries to capture molecules with good oral bioavailability properties. With just two cut-offs that focus strictly on oral bioavailability, it filters out 8% of the dataset. Another bioavailability and membrane permeability filter is the Egan filter which filters out 10% of the dataset. The molecules score lower in average descriptor values across all the examined descriptors, with both the Egan and Veber filters supporting the notion that smaller compounds are more membrane permeable and show greater bioavailability. The Mozziconacci filter, a filter for drug-like properties, applies five descriptor cutoff rules. All five descriptors used are different from the classical Rule-of-5 descriptors. The Lipinski Rule-of-5 is a set of four rules (logP, MW, and H-bond donor and acceptor cut-offs) for drug-likeness and oral bioavailability derived from a subset of 2245 drugs. It removes a similar share of the data set as well with the Lipinski filter removing 9% and the Mozziconacci filter 10%. Despite both being drug-like filters placing the filters in a chain-like matter, with the Mozziconaci filter placed after Lipinski, we filter out an additional 9% of the total dataset. This means that the drug-like definition of both filters is very different and may be used in conjunction for strict drug-like filtering. Despite only two descriptor rules for the passing of the blood–brain barrier, the Van de Waterbeemd filter removes 35% of the molecules from the database, in large part due to the small TPSA cutoff value, which is reflected in a reasonably low average TPSA descriptor value (Figure 2).The Murcko filter, due to its specificity (determining compounds with central nervous system (CNS) activity), filters out 71% percent of the database using five cut-offs. Low descriptor values for TPSA and molecular weight can also be observed as with the Veber and Egan filters, since these molecules must be smaller in order to pass the blood–brain barrier [33].

## 4. Materials and Methods

To facilitate open access use of various filters for drug design, we decided to implement the described filters into a single unit, where researchers could access various filters or combine them to a multi-filter to speed up their own drug development efforts. The first step incorporated a thorough search of the literature for information on molecular filters with the aim of defining, implementing, and sorting them as clearly as possible for the end user. Filters described were sorted into one of the two groups; filters that filter out based on the presence of functional groups and filters that filter out based on physiochemical properties.

Filters designed to exclude compounds based on the presence of functional groups most often aim to remove compounds that are reactive toward protein targets. The most common such functional groups are Michael acceptors, ketones, aldehydes, and suicide inhibitors. Such compounds would likely be false HTS positives and would increase time and money expenses spent on screening. Removing reactive functionality is based on the premise that covalent interactions are not desired for drug design except for specific cases [15]. Besides filtering for compounds with reactive species, functional group filters aim to remove optically interfering components, aggregators, fluorescent compounds, firefly luciferase inhibitors, redox cycling compounds, oxidizers, cytotoxic compounds, compounds with quenching ability, and surfactant-like compounds, all of which would frequently appear as false positives in the screening tests. Several filters fall under this category, with their properties described in Table 1 [34,35]. Some filters, although classified as functional group filters, do possess some additional property filters making them hybrid filters. We collected all filters present in the literature and added a brief description with the cut-off values on which the filter is based (Table 1 and Table 2).

The other group of filters consists of classical property filters designed to bias the chemical space of filtered libraries into a predetermined and desired direction. As stated above, the majority of such filters aim to define and narrow the scope of the library towards the drug-like paradigm. Property filters eliminate the extrema of undesired properties present in the libraries [1]. The extrema are determined from distributions in databases of desired compounds (e.g., databases of approved drugs).

**Figure 7:**
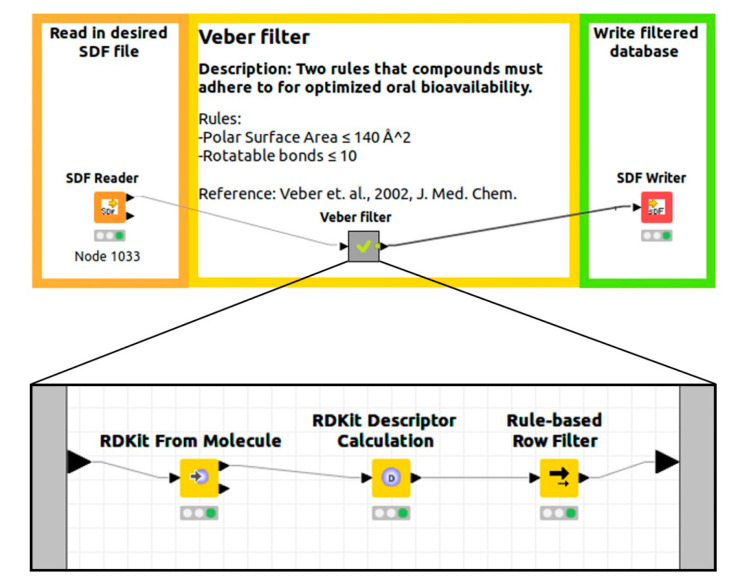
KNIME workflow example of our Veber filter implementation for effective design of compound libraries. Black lines represent the expanded meta node that contains sub-nodes [51].

After a careful analysis of the primary filter literature and the implementation of filters in existing bioinformatics software packages, KNIME was chosen as an open and accessible platform for the implementation of examined filters. Its intuitive workflow design, supported by a graphical interface, and its ability for large scale HTVS with the KNIME server makes it perfect for the integration in the established drug design workflows of users, be it ligand or structure-based drug design. KNIME allows users to create visual data flows, or pipelines, where data traverse multiple user-selected nodes. These nodes represent an essential part of KNIME, with each node possessing unique data processing capabilities, where the input and output of each node can transparently be analyzed (Figure 7) [14].

**Figure 8:**
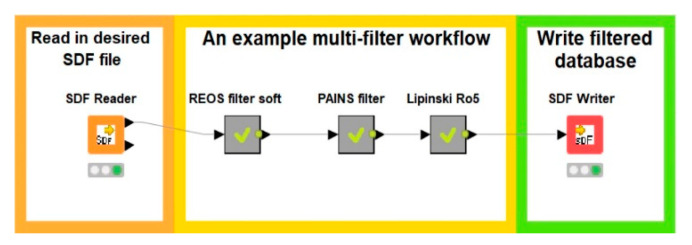
An example of combining meta nodes to form a complex drug design workflow.

The workflows were created using KNIME version 4.2.3 (available at http://knime.org accessed on 17 November 2020). Additional expansion nodes from RDKit, MOE extensions, and Vernalis KNIME were used for the final version of the workflow alongside the default KNIME nodes. All the mentioned nodes are distributed as KNIME community extensions accessible to everyone in their full functionality. All nodes and workflows are open and editable by the user if he/she wishes to change certain parameters or develop novel filters. Experienced users can expand the meta nodes and delete redundant steps in the process (e.g., duplicate generation of the canonical SMILES in the linked workflow) when combining several filters for their drug design, which would result in even faster workflows (Figure 8). The node output can be edited to produce various outputs ranging from text and table formats to chemical library formats suitable for further drug design.

We implemented 11 filters (REOS, PAINS, Aggregators, Rule-of-5, Rule-of-4, Rule-of-3, Veber filter, Mozziconacci filter, Egan filter, Van de Waterbeemd filter, Murcko filter) into our multi-filter KNIME workflow accessible at public repository (https://gitlab.com/Jukic/knime_medchem_filters/ accessed on 15 January 2022). The PAINS and REOS filter are both based on the RDKit substructure counter and compare the substructures present in the input database with a list of problematic functional groups. A rule-based row filter removes the hits from the database. The aggregation propensity detection filter, called the “aggregator filter”, evaluates the aggregation propensity based on the similarity calculated by Tanimoto coefficients of given molecules to a database containing known aggregators [15]. The user can personally control how strict the filter is with the low, medium, and high propensity filters provided. The remaining filters are knowledge-based rule-based filters that, when expanded, can often be modified by the user to suit his or her own needs. The filters are simple property counting filters that firstly calculate descriptor values using the RDKit Descriptor calculator node or the molecule properties (Mozziconacci) and then employ the rule-based row filters. The exception being the Rule-of-5 which allows one rule break, to incorporate the filter consisting of rule engines that assign the value of 1 for each rule break, with the math formula summing up all the values and the final rule-based row filter comparing the value to see. The impact of strict cut-offs that define specific chemical spaces and milder filters such as the Lipinski’s Rule-of-5 which allow a rule break can be seen in Figure 5 and Figure 6.

## 5. Conclusions

After analyzing and implementing several molecular medicinal chemistry filters and testing the created workflows, we conclude that compound filters are essential for modern computer aided drug design (CADD). They provide the researcher with a simple, fast, and robust way to enrich the chemical space and to reduce the time associated with post-filtering methods. They are also easy to use and can be customized to particular preferences of the studied chemical space. However, the user must be aware of the properties used for filtering, as some, such as REOS and PAINS, were not designed with covalent chemistry in mind. In such cases, it is better to flag the compounds for a later evaluation. We firmly believe that this article provides medicinal chemistry community with a handful of useful workflows for novel drug design, identification, and HTVS, as well as with a good initial overview of compound filtering in drug discovery.

## Figures and Tables

**Table 1:** The most common functional group filters described in the scientific literature presented in alphabetical order.

Name/Reference	Description	Features/Cutoff Values
Aggregators [36]	Tanimoto coefficient similarity search to a database of known aggregators.	Tanimoto coefficient similarity ≥ 0.85 or SlogP > 5 (high similarity),
		Tanimoto coefficient similarity ≥ 0.5 and SlogP > 3 (medium similarity),
		Tanimoto coefficient similarity < 0.85 and SlogP ≤ 3 (low similarity)
Ely Lilly Rules [37]	A set of 275 rules, developed over an 18-year period, used to identify compounds that may interfere with biological assays, allowing their removal from screening sets.	Reasons for rejection of compounds: reactivity, interference with assay measurements (fluorescence, absorbance, quenching), instability and lack of druggability (lacking both oxygen and nitrogen)
Muegge method [18,38]	Bioavailability prediction rules dubbed the Muegge method. Pharmacophore filter developed by analyzing known drug databases, with four functional molecular motifs determined to be important in drug-like molecules:	Primary, secondary, and tertiary amines are considered pharmacophore points but not pyrrole, indole, thiazole, isoxazole, other azoles, or diazines. Compounds with more than one carboxylic acid are dismissed. Compounds without a ring structure are dismissed. Intracyclic amines that occur in the same ring are fused and count as only one pharmacophore point.
PAINS [39]	Removal of frequent hitters (promiscuous compounds) by identifying sub-structural features not recognized by filters commonly used to identify reactive compounds.	Functional groups such as rhodanines, phenolic Mannich bases, hydroxyphenylhydrazones, alkylidene barbiturates, alkylidene heterocycles, 1,2,3-aralkylpyrroles, activated benzofurazans, 2-amino-3-carbonylthiophenes, catechols, and quinones do not pass the filters.
REOS ^1^ [3,40,41]	Seven property filters	H-bond donor ≤ 5,
	(similar to the PATTY	H-bond acceptors ≤ 10,
	rules in program developed at Merck)	−2 ≤ Formal charge ≤ +2,
		Number of rotatable bonds ≤ 8,
		200 ≤ Molecular weight ≤ 500,
		20 ≤ number of heavy atoms ≤ 50,
		−2 ≤ logP ≤ 5
	Functional group filters for the removal of problematic structures dubbed REOS (rapid elimination of swill; program developed at Vertex).	Reactive, toxic and other undesirable moieties such as nitro groups, preoxides, triflates, aldehydes, acetals, etc.

^1^ REOS is a hybrid filter which combines a set of functional group filters with property filters. As the REOS filter can be combined with other (property) filtering schemes, the property filtering part can be omitted and only functional group filters employed. As implemented in KNIME, the user can also specify the maximum quantity for each of the functional group rules, tuning the filter to the needs of the individual research scenario. REOS moieties in the SMARTS format can be found inside the KNIME workflow “REOS substructures” node.

**Table 2:** The most common property filters described in the scientific literature.

Name/Reference	Description	Features/Cutoff Values
Egan [42]	Set of rules designed by analyzing the data on compounds both well and poorly absorbed in humans with multivariate statistics. Two descriptors (AlogP and PSA) were chosen for inclusion when determining membrane permeability. Compounds that pass exhibit good bioavailability.	AlogP ≤ 5.88,
		polar surface area ≤ 131.6 Å^2^
Fichert [43]	Rules for structure-permeability based on a set of 41 small drug-like molecules. LogD is the main property that determines permeability, with structures passing this filter being highly permeable in the Cacao-2 model.	Molecular weight ≤ 500,
		0 ≤ logD ≤ 3
Ghose [44]	A set of rules for drug-likeness derived from characterizing 6304 compounds taken from the Comprehensive Medicinal Chemistry Database.	180 ≤ molecular weight ≤ 480,
		40 ≤ molecular refractivity ≤ 130,
		−0.4 ≤ ClogP ≤ 5.6,
		20 ≤ number of atoms ≤ 70
Lee filter [45]	Analysis of natural products to determine potential appealing scaffolds for future drug design. Pharmacophoric properties of natural products, trade drugs, and virtual combinatorial library were assessed, finding key properties and several scaffolds which could work as building blocks.	MW mean ~356
		LogP mean ~2.1
Lipinski (Rule-of-5) [13]	A set of four rules for drug-likeness and oral bioavailability derived from a subset of 2245 drugs from the World Drug Index. The rules aim to address the ADME issues.	Molecular weight ≤ 500,
		logP ≤ 5,
		H-bond donors ≤ 5,
		H-bond acceptors ≤ 10
Mozzicconacci [46]	Filter developed by Mozziconacci after analyzing 15 freely available chemical libraries (2 million compounds). Drug-likeness was examined using common chemical features and based on the successive filters were designed to extract the drug-like subset.	Rotatable bonds ≤ 15,
		number of rings ≤ 6,
		oxygen atoms ≥ 1,
		nitrogen atoms ≥ 1,
		halogen atoms ≤ 7
Murcko filter [33,47]	Rules for determining CNS activity, joining 7 property descriptors (Rule-of-5 with the addition of rotatable bonds, aromatic density, and a measure for branching) and 166 fingerprint descriptors to determine presence or absence of functional groups.	MW 200–540,
		logP 0–5.2,
		H-bond acceptors ≤ 4,
		H-bond donor ≤ 3,
		rotatable bonds ≤ 7,
		branching behavior 3.4–12.2,
		aromatic rings < 3
Oprea Lead-Like [1,24]	A set of rules based on lead-like vs. drug-like comparison after examination of several commercially available databases. The rules aim to maintain focus towards effective and orally absorbable compounds. Beside the properties chosen based on the Rule-of-5, additional properties were chosen to better reflect molecular complexity of a library and the rigidity of a molecule.	Molecular weight < 450,
		−3.5 ≤ logP < 4.5,
		−4 ≤ logD ≤ 4,
		number of rings ≤ 4,
		nonterminal single bonds ≤ 10,
		H-bond donor ≤ 5,
		H-bond acceptor ≤ 8
Rule-of-3 [48]	Rules designed to support “fragment-based” drug research. Hits obtained using this filter can be useful for fragment libraries used to generate potential leads. Fragment libraries are useful for sampling chemical diversity or targeting specific interactions.	Molecular weight ≤ 300,
		logP ≤ 3,
		H-bond donor ≤ 3,
		H-bond acceptors ≤ 3
		rotatable bonds ≤ 3
Rule-of-4 [19]	A set of rules derived from analyzing the 2P2I database that contains protein–protein interaction inhibitors with the aim of establishing guidelines for druggable protein–protein inhibitors, since these most often break traditional property filter rules.	Molecular weight ≥ 400,
		logP ≥ 4,
		number of rings ≥ 4,
		H-bond acceptors ≥ 4
van de Waterbeemd [49,50]	Physiochemical properties for estimation of blood–brain barrier crossing of compounds. Rules were derived by examination of lipophilicity, H-bonding capacity, and molecular shape and size descriptors of marketed CNS and CNS-inactive drugs.	Molecular weight ≤ 450,
		polar surface area ≤ 90 Å^2^
Veber [51]	Two rules to meet the criteria for oral bioavailability derived after studying bioavailability measurements in rats for of over 1100 drug candidates at GlaxoSmithKline.	Rotatable bonds ≤ 10,
		polar surface area ≤ 140 Å^2^

## Data Availability

Not applicable.

## Abbreviations

CC	combinatorial chemistry
HTS	high-throughput screening
VS	virtual screening
HTVS	high-throughput virtual screening
ADME	absorption, distribution, metabolism, and excretion
MW	molecular weight
Ro4	Rule-of-4
Ro5	Rule-of-5
Ro3	Rule-of-3
SDF	structure-data file format
MOL	MDL Molfile
SMILES	simplified molecular input line entry specification format
SMARTS	SMILES arbitrary target specification
KNIME	Konstanz Information Miner
SD	standard deviations
SMR	molecular refractivity
TPSA	total polar surface area
MW	molecular weight
HBA	No. of hydrogen bond acceptors
HBD	No. of hydrogen bond donors
REOS	rapid elimination of swill
PAINS	pan-assay interference compounds
CNS	central nervous system
